# Dental Management of Ectodermal Dysplasia: A Report of Two Clinical Cases

**DOI:** 10.7759/cureus.84031

**Published:** 2025-05-13

**Authors:** Fatima Ezzahra Elgasmi, Meryem Rahmaoui, Samira Elarabi, Bouchra Badre

**Affiliations:** 1 Department of Pediatric Dentistry, Faculty of Medicine, Pharmacy and Dental Medicine, Sidi Mohamed Ben Abdellah University, Fez, MAR; 2 Department of Pediatric Dentistry, Mohammed VI National Center for the Disabled in Casablanca, Casablanca, MAR; 3 Department of Pediatric Dentistry, Faculty of Dental Medicine, Hassan II University of Casablanca, Casablanca, MAR; 4 Department of Pediatric Dentistry and Laboratory of Community Health, Epidemiology and Biostatistics, Faculty of Dental Medicine, Hassan II University of Casablanca, Casablanca, MAR

**Keywords:** dental rehabilitation, ectodermal dysplasia, hypodontia, pediatric dentistry, removable prosthesis

## Abstract

Ectodermal dysplasia (ED) represents a group of hereditary disorders affecting ectoderm-derived structures, including teeth, hair, nails, and sweat glands. Dental anomalies, such as hypodontia, anodontia, and conical-shaped teeth, are hallmark features of this condition. This article presents two pediatric cases of hypohidrotic ectodermal dysplasia, each exhibiting distinct oral and functional manifestations. We discuss the clinical presentation, radiographic features, and individualized, multidisciplinary dental management strategies used for each child. Early diagnosis and intervention are essential to improve oral function, aesthetics, and psychosocial outcomes.

## Introduction

Ectodermal dysplasias (EDs) are a heterogeneous group of hereditary disorders characterized by abnormalities in at least two ectoderm-derived structures, including hair, nails, sweat glands, and teeth [1]. Among its various forms, hypohidrotic ectodermal dysplasia (HED) is the most common, typically inherited in an X-linked recessive pattern [2]. The classic clinical triad includes hypodontia or anodontia, hypotrichosis, and hypohidrosis, though the severity of manifestations can vary considerably [3].

Dental anomalies often serve as the first indication of ED, particularly in pediatric patients. These may include missing teeth, conical-shaped crowns, delayed eruption, and the underdevelopment of the alveolar ridge. Such anomalies significantly impact oral function, facial growth, speech development, and psychosocial well-being [4,5]. The prevalence of ED is estimated at approximately 1 in 100,000 live births, although this number may be underestimated due to variable expression [6].

## Case presentation

Clinical case 1

A 3-year-old male child was brought to the pediatric dentistry department with complaints of missing teeth and difficulty in chewing solid foods. According to the parents, the child had shown signs of ectodermal dysfunction since infancy, including dry skin, absence of sweating, and repeated episodes of overheating. By age two, he had not developed any visible teeth and struggled to consume solid foods. There were also early signs of delayed speech development and self-consciousness in social settings due to orofacial appearance. Notably, the child’s younger brother presented similar signs and was later diagnosed with the same condition. A positive history of consanguinity was also reported.

**Figure 1:**
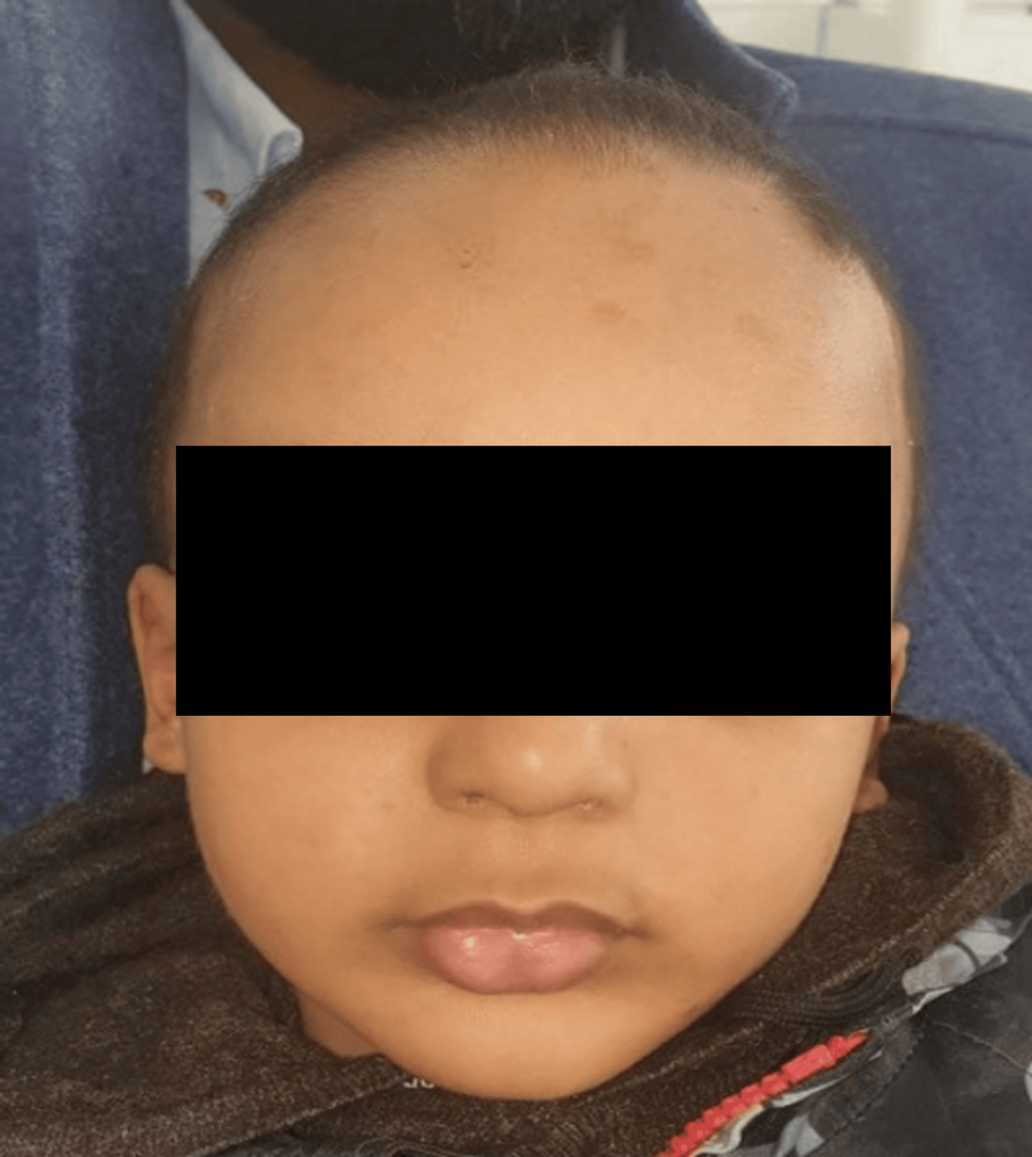
Extraoral frontal view showing sparse hair, prominent forehead, and dry skin A frontal extraoral photograph of the three-year-old male patient showing typical facial features of hypohidrotic ectodermal dysplasia, including sparse hair, dry skin, and everted lips.

Extraoral examination revealed classic features of hypohidrotic ectodermal dysplasia, including sparse scalp hair, fine eyebrows, dry skin, a depressed nasal bridge, prominent frontal bossing, and everted lips. The child’s skin was xerotic and appeared sensitive to heat. His general physical and psychomotor development was otherwise within normal limits (Figure 1).

**Figure 2:**
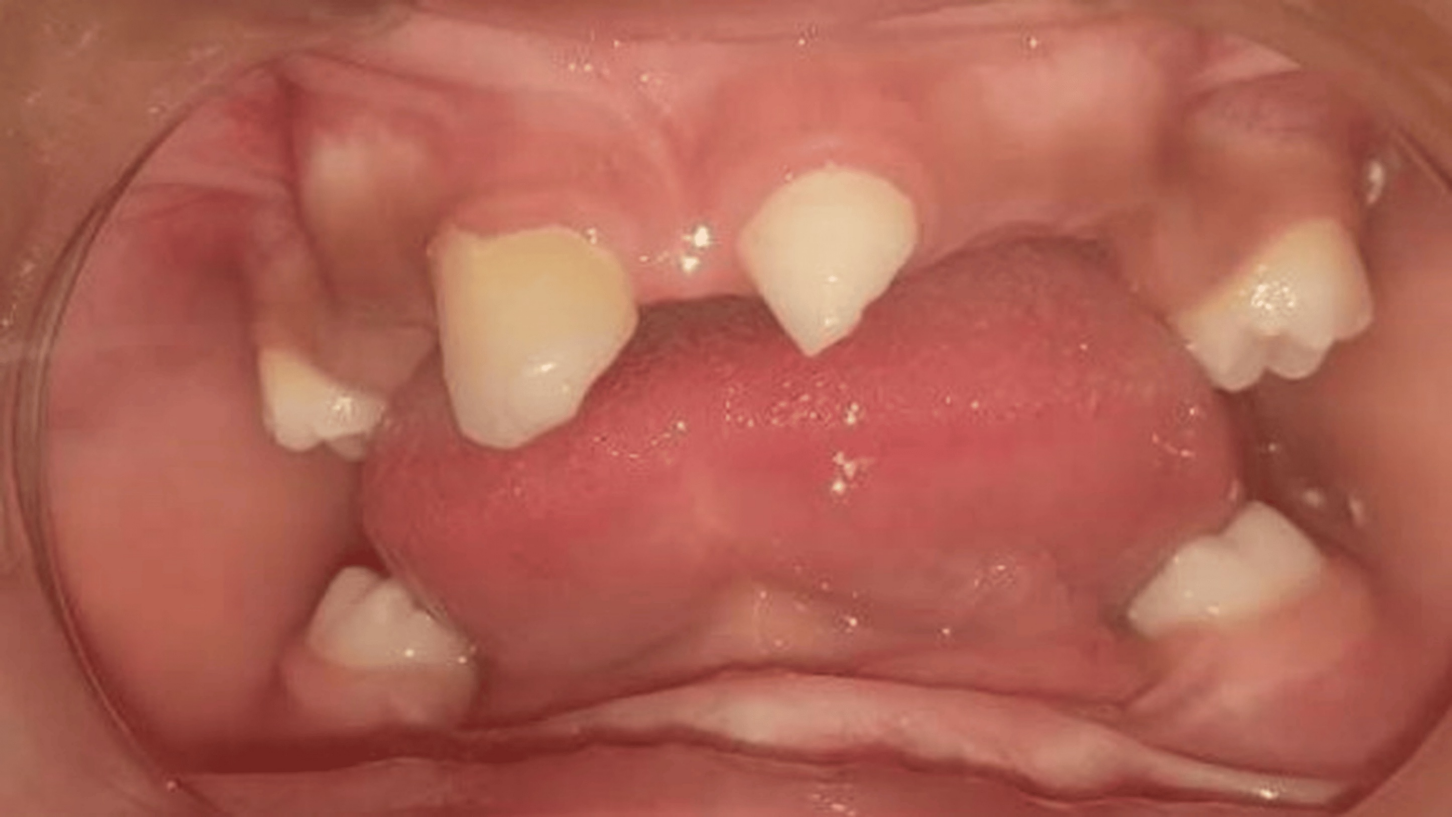
Intraoral view showing a U-shaped arch, resorbed ridges, and conical anterior teeth An intraoral view revealing a U-shaped maxillary arch and a resorbed knife-edge mandibular ridge, with only two conical primary maxillary central incisors present.

An intraoral examination revealed a severely resorbed and thin mandibular alveolar ridge with a U-shaped maxillary arch. Only two maxillary primary central incisors (teeth 51 and 61) were present, both conical in shape. Although some primary molars were clinically visible, they were morphologically normal and asymptomatic, and therefore not considered diagnostically significant in the context of ectodermal dysplasia. The oral mucosa appeared dry, and salivary flow was reduced (Figure 2).

**Figure 3:**
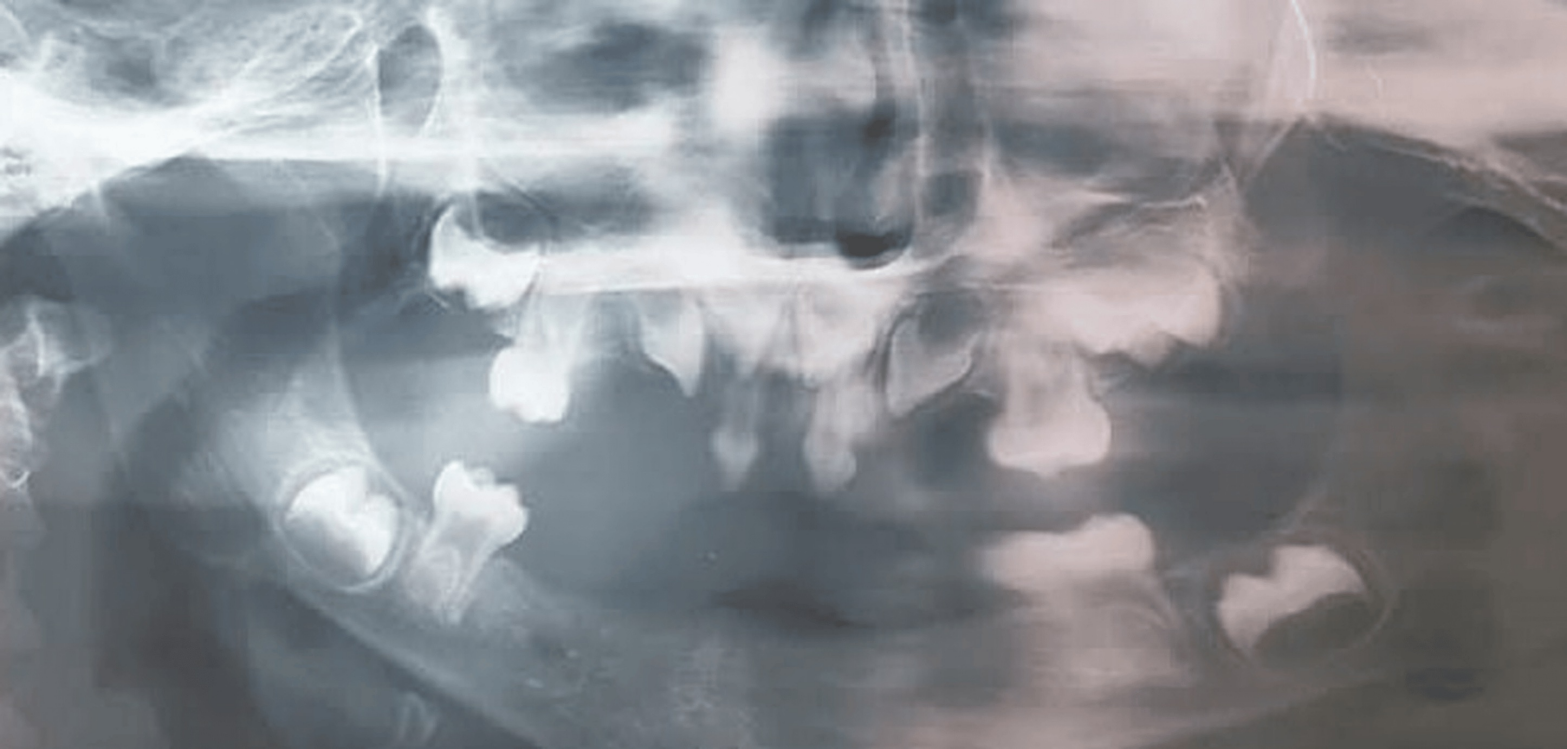
Panoramic radiograph showing oligodontia and conical permanent tooth buds A panoramic radiograph showing multiple congenitally missing teeth, conical-shaped anterior teeth, and the presence of four second primary molars with early permanent molar buds.

A panoramic radiograph showed the presence of four second primary molars (55, 65, 75, and 85) with early development of the first permanent molars and maxillary central incisors. The mandibular alveolar ridge was markedly underdeveloped in the anterior region (Figure 3).

Based on clinical and radiographic findings, a diagnosis of hypohidrotic ectodermal dysplasia with severe functional and esthetic implications was established.

Treatment consisted of behavioral management and oral hygiene instruction. The four erupted second primary molars (teeth 55, 65, 75, and 85) were preserved with preventive and functional care, including selective grinding and fluoride application, as they were healthy and contributed to prosthetic support.

The two conical maxillary primary central incisors (teeth 51 and 61) were reshaped using direct composite resin build-ups to approximate the anatomy of natural incisors and improve esthetics and phonetics.

**Figure 4:**
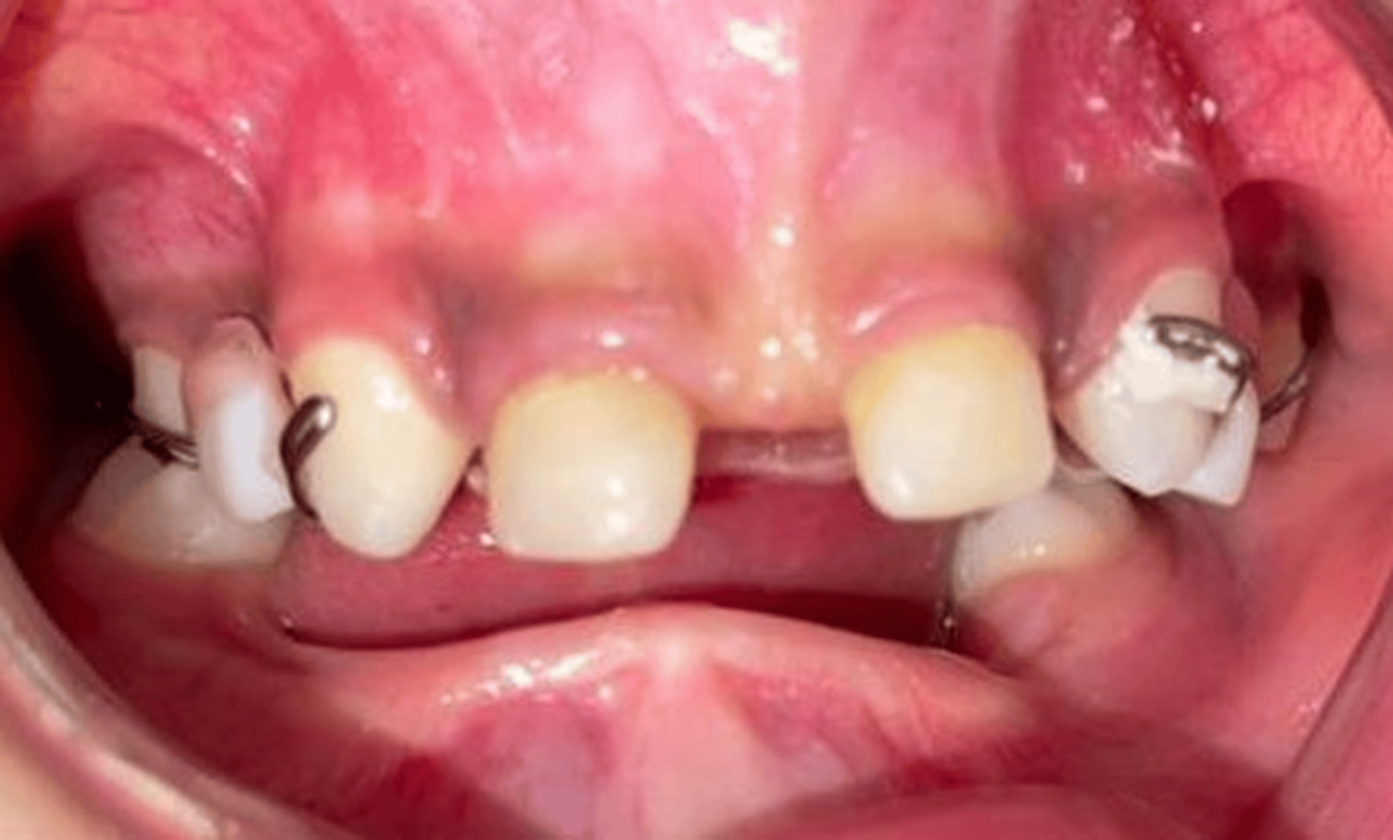
Intraoral view showing anterior composite build-ups and prosthetic retention An intraoral photograph showing composite resin build-ups on the maxillary anterior teeth, restoring proper crown morphology and aesthetics, with orthodontic wire retainers ensuring stability of the partial prosthesis.

**Figure 5:**
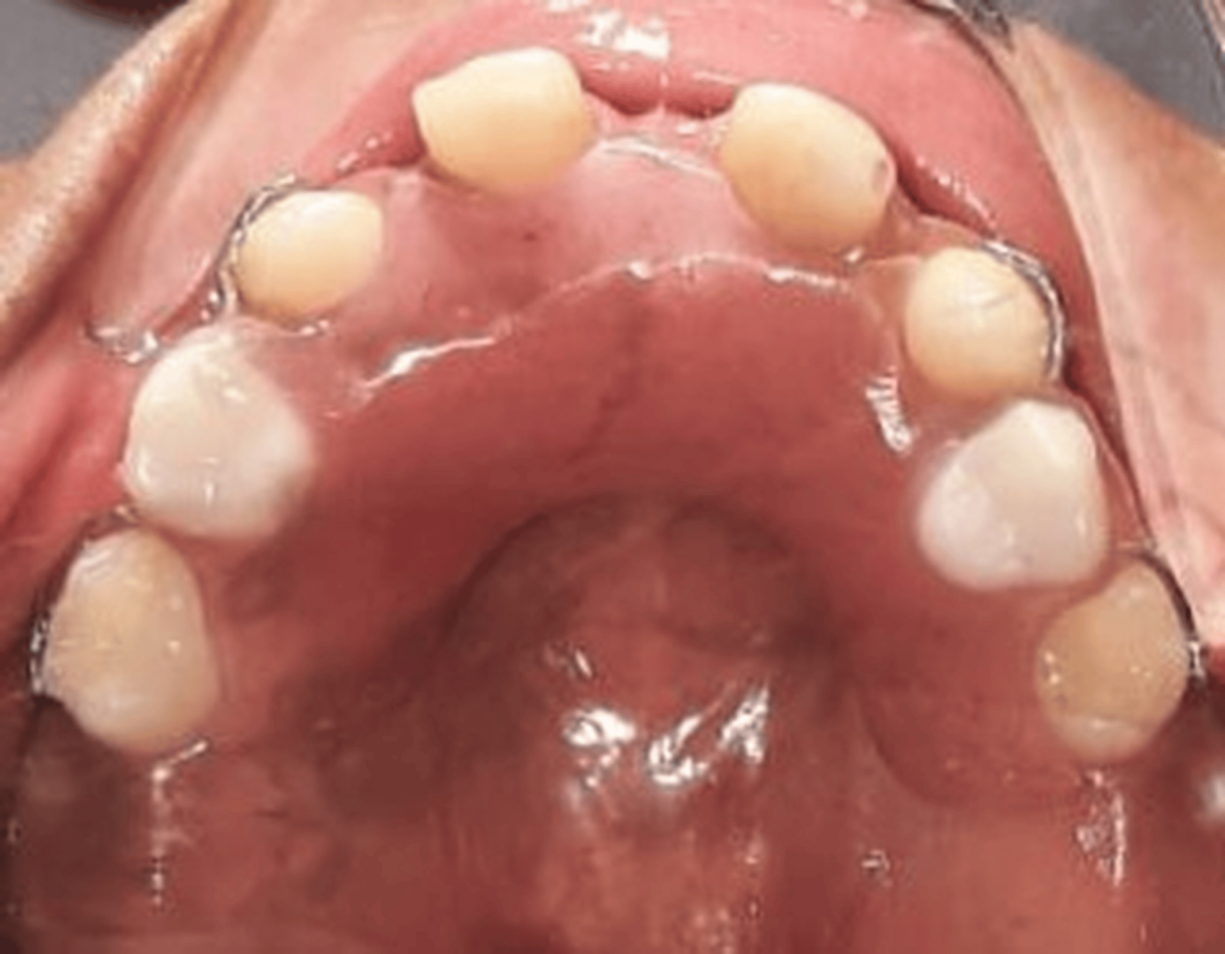
Intraoral view of the maxillary arch with removable prosthesis and orthodontic components An intraoral photograph demonstrating a maxillary removable partial denture with orthodontic clasps retaining prosthetic anterior teeth, restoring anterior esthetics and arch integrity.

**Figure 6:**
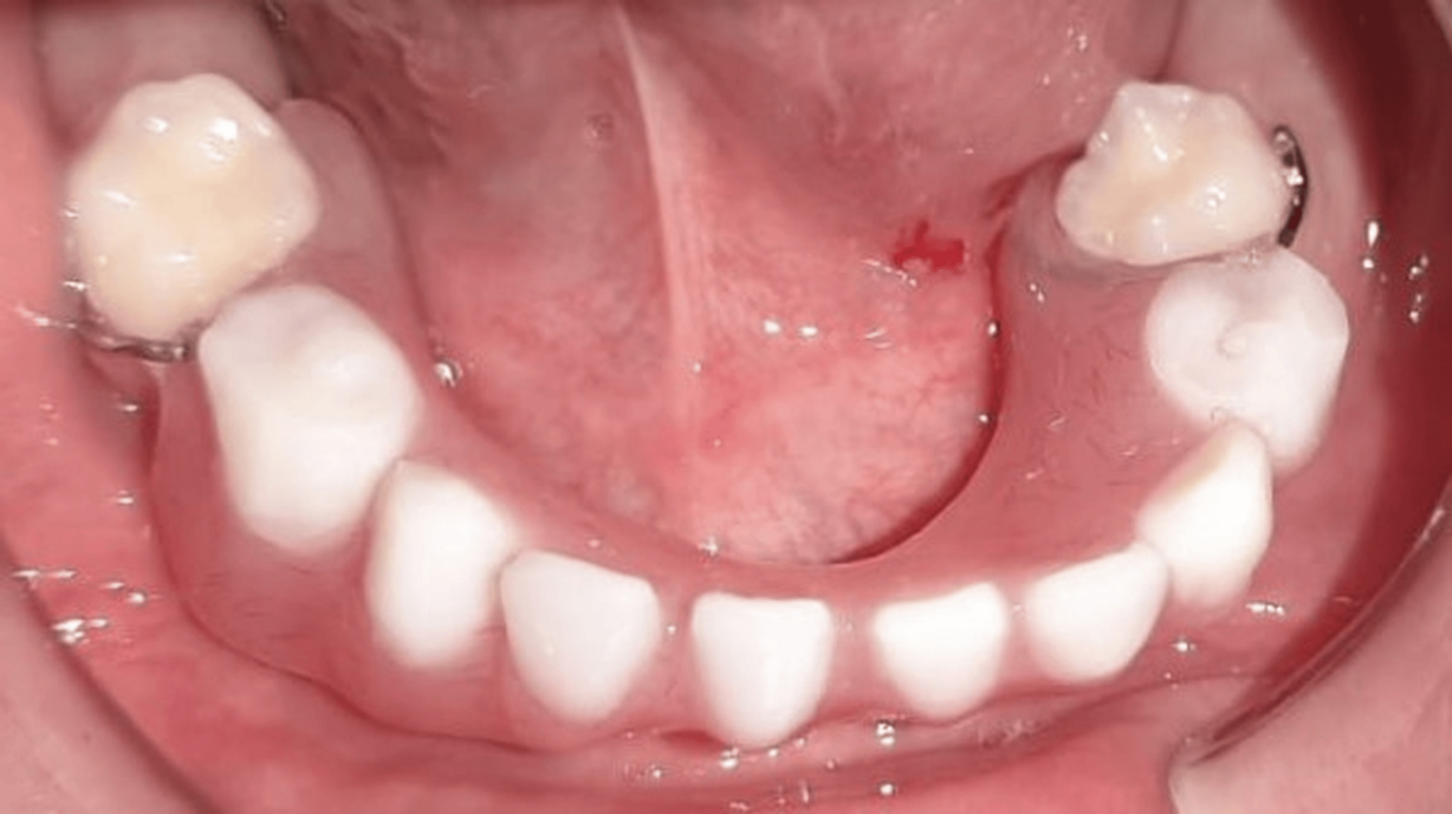
Intraoral view of the mandibular arch after prosthetic rehabilitation An intraoral photograph showing a complete lower removable prosthesis in place, restoring masticatory function and occlusal vertical dimension.

Maxillary and mandibular complete removable dentures were fabricated to restore masticatory function, speech, vertical dimension, and facial harmony. Jaw relations were recorded using wax rims, and the occlusion was carefully adapted to the child’s needs and growth pattern. The prostheses were delivered and adjusted in multiple sessions to ensure comfort, retention, and functional integration (Figures 4-6).

Post-treatment follow-up was intensive during the adaptation period, initially on a weekly basis, then monthly for the first six months. Thereafter, the patient was scheduled for check-ups every six months to assess prosthetic fit, oral hygiene, and craniofacial development. As the child grew, new prostheses were fabricated to accommodate changes in arch size, vertical dimension, and occlusal relationships.

Clinical case 2

A 10-year-old female patient was referred to the pediatric dentistry department for evaluation of missing teeth and dissatisfaction with her dental appearance. According to the parents, the child had experienced episodes of hyperthermia and dry skin during infancy. Dental anomalies became evident around the age of six, when several permanent teeth failed to erupt, and the erupted teeth appeared abnormally shaped. Notably, the patient’s younger brother presented similar signs and was also diagnosed with ectodermal dysplasia. A positive history of consanguinity was reported.

**Figure 7:**
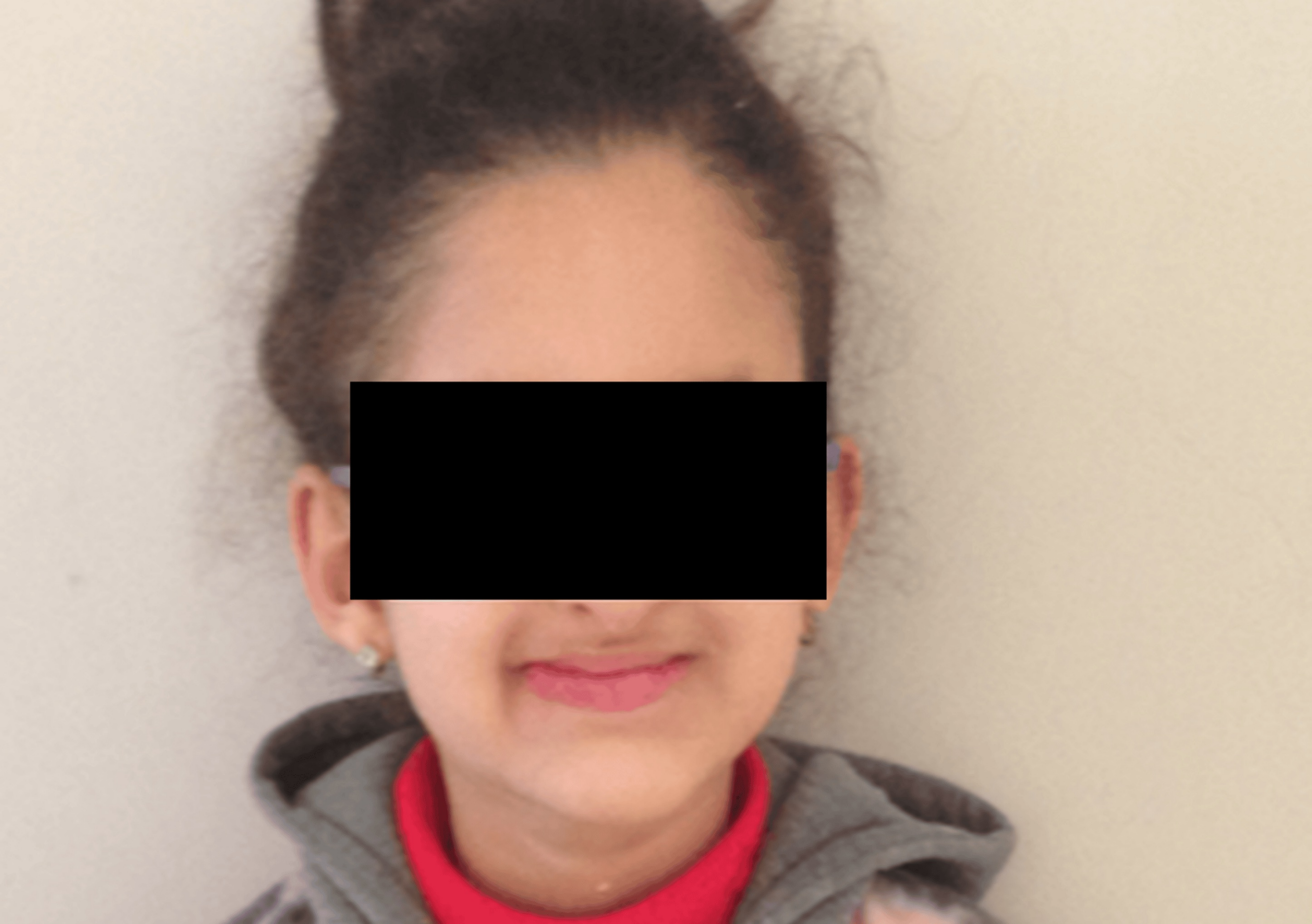
Extraoral frontal view showing mild facial features of ectodermal dysplasia A frontal extraoral photograph of the 10-year-old female patient showing mild signs of ectodermal dysplasia, including dry skin.

**Figure 8:**
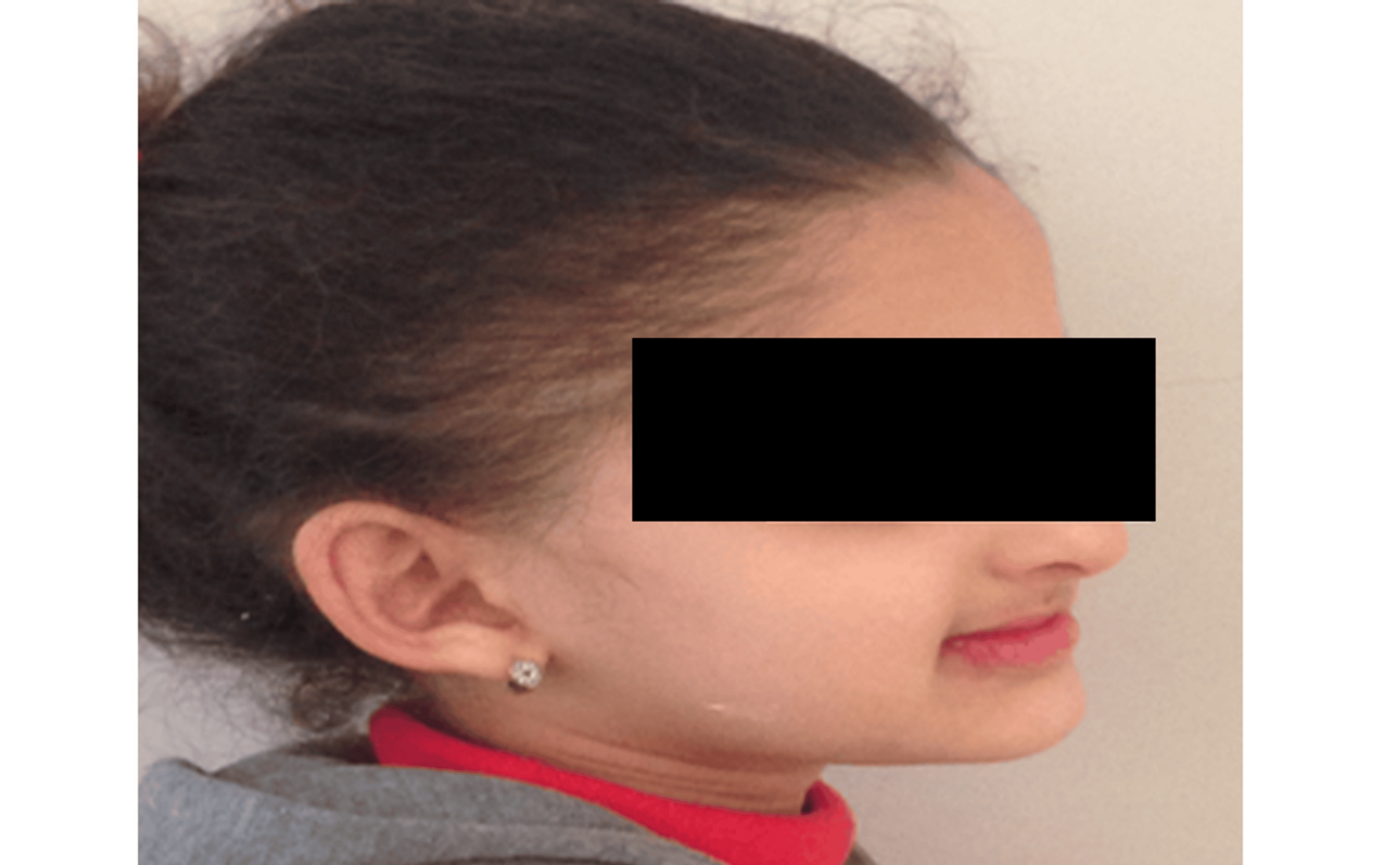
Extraoral profile view of the patient A lateral profile of the same patient highlighting soft facial features and reduced lip support due to dental agenesis.

Extraoral examination revealed mild ectodermal features, including sparse eyebrows, dry skin, and frontal bossing. The patient’s profile showed retruded lips and a flattened midface. Psychosocially, the patient avoided smiling and had developed low self-esteem related to her dental appearance (Figure 7 and Figure 8).

**Figure 9:**
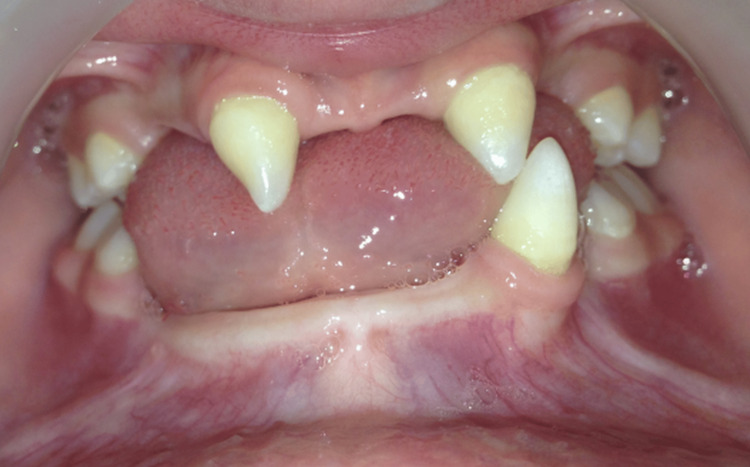
Intraoral image showing conical permanent anterior teeth and partial hypodontia An intraoral view showing conical-shaped permanent maxillary incisors and agenesis of multiple teeth.

An intraoral examination showed multiple missing permanent teeth, with the presence of conical-shaped permanent maxillary lateral incisors. The first permanent molars (teeth 16, 26, 36, and 46) were present, erupted, and exhibited normal morphology. The mandibular and maxillary ridges were moderately developed. Four second primary molars (55, 65, 75, 85) were still present, caries-free, and functionally stable. The oral mucosa appeared dry but healthy (Figure 9).

**Figure 10:**
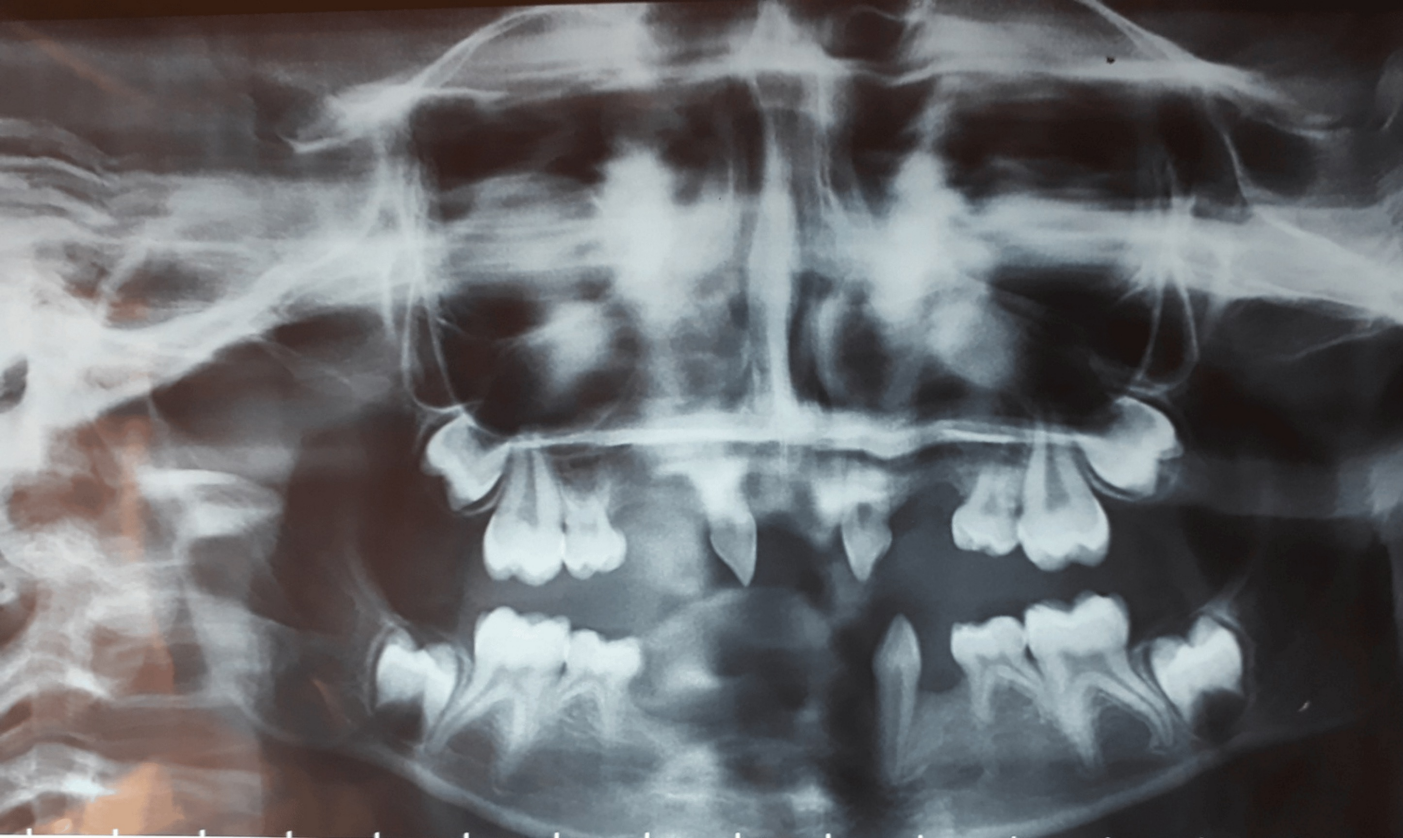
Panoramic radiograph showing agenesis of multiple permanent tooth buds A panoramic radiograph revealing multiple missing permanent tooth buds and conical morphology of erupted teeth.

A panoramic radiograph confirmed the absence of several permanent tooth buds and the persistence of primary molars (Figure 10).

Based on the clinical and radiographic findings, a diagnosis of hypohidrotic ectodermal dysplasia with partial hypodontia was established.

Treatment included preventive and functional care of the four retained primary molars (55, 65, 75, 85) to preserve them as long as possible for support and function. The anterior conical permanent teeth were preserved in their original form and used as abutment support for the removable partial prosthesis; no restorative build-up was performed.

**Figure 11:**
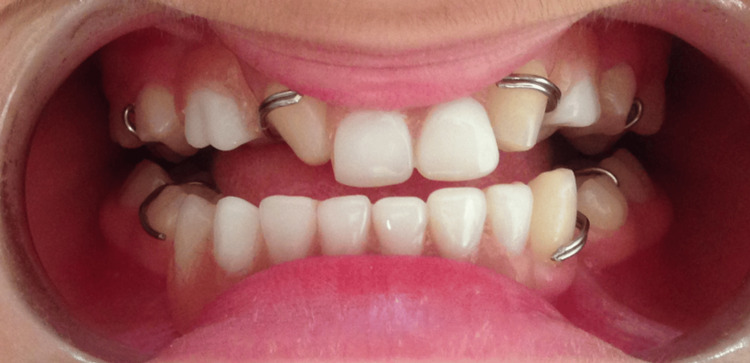
A post-treatment view after removable partial denture insertion A post-treatment intraoral image showing the removable partial denture in place, providing improved aesthetics, occlusal support, and function.

A removable partial prosthesis was fabricated for the maxillary arch to replace the missing teeth, improve function and aesthetics, and provide lip support. Jaw relations were recorded using wax rims, and the prosthesis was designed to respect occlusal balance and patient comfort. The appliance was adjusted progressively to ensure optimal retention and adaptation (Figure 11).

The patient was followed weekly during the initial adaptation phase, then monthly during the first six months, and then every six months. The prosthesis was remade periodically to accommodate changes in jaw growth, occlusion, and functional needs. Psychological support and positive reinforcement were also part of the follow-up to encourage self-confidence and oral hygiene compliance.

## Discussion

Ectodermal dysplasia presents a wide clinical spectrum that requires early, individualized management, particularly in pediatric patients. In the first case, a three-year-old male presented with severe oligodontia, conical teeth, and complete anodontia in the anterior mandible. Such early and severe forms are typical of X-linked hypohidrotic ectodermal dysplasia and are frequently associated with significant impairments in mastication, speech development, and psychosocial integration [3,4]. Early prosthetic intervention, such as complete removable dentures, has been shown to restore oral function and support facial growth [7]. Schnabl et al. emphasized that timely prosthetic rehabilitation contributes to improved maxillofacial development and helps reduce social distress in affected children [8].

The second case, a 10-year-old female, displayed a milder phenotype with partial hypodontia and conical anterior teeth. In such cases, management often combines conservative composite build-ups and removable partial dentures. Kaushik et al. and Shamim et al. advocate for such age-appropriate and minimally invasive strategies to optimize function and esthetics without compromising future treatment options [7-11]. The application of direct composite restorations in children with ED has also been shown to enhance esthetics, masticatory efficiency, and self-esteem [9].

Both patients in this report benefited from early psychological support, an often underemphasized aspect of care. As Mittal et al. and Joseph et al. have shown, the psychosocial impact of ED, particularly in school-aged children, is profound and can affect social interaction and academic engagement [12,13]. Multidisciplinary coordination is therefore crucial not only for functional rehabilitation but also for the child’s emotional development.

Standard therapeutic protocols for ED typically begin with early prosthetic rehabilitation using removable complete or partial appliances to restore masticatory function and facial harmony [3,5]. These appliances are regularly modified or replaced in response to craniofacial growth. Definitive interventions, such as dental implants, are deferred until skeletal maturity is achieved to ensure long-term success. Studies by Cezária et al. and AlNuaimi et al. have reported successful implant-supported prosthetic rehabilitation in adolescent ED patients with adequate bone development, reinforcing the importance of longitudinal planning and follow-up [6,10].

## Conclusions

The clinical management of ectodermal dysplasia in pediatric patients requires a tailored and growth-adapted approach. In both cases presented, early prosthetic rehabilitation, whether complete or partial, combined with conservative restorative techniques and psychological support, significantly improved oral function, appearance, and social confidence. The preservation of strategic primary teeth, reshaping of conical anterior teeth, and timely delivery of removable prostheses allowed for functional and esthetic outcomes compatible with the patients’ developmental stages. These cases underscore the value of early diagnosis, multidisciplinary coordination, and progressive follow-up in optimizing both clinical results and quality of life in children affected by ectodermal dysplasia.
